# Degradation of lignin in different lignocellulosic biomass by steam explosion combined with microbial consortium treatment

**DOI:** 10.1186/s13068-023-02306-2

**Published:** 2023-03-30

**Authors:** Wen Zhang, Chenyang Diao, Lei Wang

**Affiliations:** 1 College of Biology and Environmental Engineering, Zhejiang Shuren University, Hangzhou, 310015 Zhejiang China; 2 Key Laboratory of Pollution Exposure and Health Intervention of Zhejiang Province, Zhejiang Shuren University, Hangzhou, 310015 Zhejiang China; 3 School of Engineering, Westlake University, Hangzhou, 310024 Zhejiang China; 4 Institute of Advanced Technology, Westlake Institute for Advanced Study, Hangzhou, 310024 Zhejiang China

**Keywords:** Lignin degradation, Microbial consortium, Biomass pretreatment

## Abstract

The difficulty of degrading lignin is the main factor limiting the high-value conversion process of lignocellulosic biomass. The biodegradation of lignin has attracted much attention because of its strong environmental friendliness, but it still faces some dilemmas such as slow degradation rate and poor adaptability. The microbial consortia with high lignin degradation efficiency and strong environmental adaptability were obtained in our previous research. To further increase the lignin degradation efficiency, this paper proposes a composite treatment technology of steam explosion combined with microbial consortium degradation to treat three kinds of biomass. We measured the lignin degradation efficiency, selectivity value (SV) and enzymatic saccharification efficiency. The structural changes of the biomass materials and microbial consortium structure were also investigated. The experimental results showed that after 1.6 MPa steam explosion treatment, the lignin degradation efficiency of the eucalyptus root reached 35.35% on the 7th days by microbial consortium. At the same time, the lignin degradation efficiency of the bagasse and corn straw treated by steam explosion followed by microbial biotreatment was 37.61–44.24%, respectively, after only 7 days of biotreatment. The microbial consortium also showed strong selectivity degradation to lignin. The composite treatment technology can significantly improve the enzymatic saccharification efficiency. Saccharomycetales, *Ralstonia* and Pseudomonadaceae were the dominant microorganisms in the biomass degradation systems. It was proved that the combined treatment technology of steam explosion and microbial consortium degradation could overcome the drawbacks of traditional microbial pretreatment technology, and can facilitate the subsequent high-value conversion of lignocellulose.

## Introduction

Due to the large consumption of fossil energy in the world, excessive greenhouse gases are emitted into the air every year. The environmental harm and climate impact caused by the increase of greenhouse gas content have been widely concerned all over the world. In 2020, China’s total carbon emission reached 10.251 billion tons, with the energy consumption and carbon dioxide emissions second only to the United States [1]. The carbon dioxide emission of China would rise to the first around 2025 [2]. Therefore, it is urgently needed to control carbon dioxide emissions and reduce the use of fossil energy at present. Many countries in the world are implementing carbon emission reduction plans. For example, China has proposed the *Dual Carbon* strategic goal of achieving carbon peak by 2030 and carbon neutrality by 2060. On the other hand, with the tension and changes in the international situation, there is a great risk of countries relying too much on fossil energy. Therefore, the efficient use of biomass energy has received extensive attention.

The efficient utilization of biomass energy can replace the consumption of some fossil energy and reduce carbon emissions [3]. Biomass will absorb carbon dioxide in the atmosphere during its growth, and biomass energy is a carbon *Zero emission* energy in the sense of life cycle analysis. Lignocellulose biomass is the most abundant renewable biomass resource on earth, with wide sources and low cost. According to the data from the Ministry of Agriculture and Rural Affairs of China, the production of straw lignocellulose in China reached 797 million tons in 2020 [4]. If effective treatment technology can be developed to convert the waste lignocellulose biomass into high-value products such as biofuel ethanol, the shortage of fossil energy can be effectively alleviated, and the carbon emission caused by lignocellulose incineration should be reduced [5]. Therefore, it is vital to develop and utilize lignocellulose biomass resources.

The raw materials of lignocellulose are mainly composed of cellulose, hemicellulose and lignin. Lignin is closely connected with hemicellulose molecules through covalent and hydrogen bonds, making lignocellulose a strong structure and highly resistant to enzymatic and microbial hydrolysis [6]. Lignin is considered as a natural barrier against the degradation of lignocellulose biomass, which brings difficulties to the industrial biorefinery of lignocellulose biomass. The degradation of lignin can improve the pretreatment efficiency of lignocellulosic biomass, realize the effective separation of cellulose, hemicellulose and lignin, and mildly convert lignocellulosic biomass into bioenergy and other valuable products [7]. Exploring and developing efficient lignin degradation technology has become a research hotspot in recent years [8].

The existing research on the efficient degradation of lignin indicates that lignin can be degraded by physical and chemical methods. However, most of these methods require harsh operating conditions or high energy-consumption equipment, and there are some problems such as inhibitors in the degradation process, affecting the subsequent enzymatic hydrolysis and fermentation process. Biological pretreatment is considered as a potential method because of its advantages of low energy consumption, little chemical addition and environmental friendliness [9].

Traditional lignin-biodegradation microorganisms are represented by fungi such as white rot fungi, but fungal degradation faces some problems, such as slow reaction process and high requirements for strain culture environment and conditions [10]. Compared with fungi, bacteria can degrade lignin in a faster way, but its degradation efficiency is lower [11]. The direct use of lignin-degrading enzymes has the disadvantages of high cost and easy inactivation. How to achieve rapid, efficient and low-cost degradation is the key problem to be solved in the industrial application of lignin microbial degradation.

The decomposition of lignin in nature is the result of the cooperation of fungi and bacteria in the natural microbial community [12]. In view of the potential strengths of microbial consortium, our previous research successfully screened four microbial consortia of J-1, J-6, J-8 and J-15 with high lignin degradation efficiency from the decayed wooden relics [13]. Among them, the lignin degradation efficiency of J-6 was highest and reached 54% after 48 h treatment with an initial lignin concentration of 0.5 g/L. Our work also demonstrated the potential utilization of microbial consortia via the synergy of fungi and bacteria, which can overcome the shortcomings of traditional lignin biodegradation when using a single strain. This indicates the potential application of the microbial consortium to lignin degradation and removal. In addition, microorganisms in the microbial consortium can form metabolic complementarity or nutrition dependence through interaction in the metabolic system, thereby driving the metabolic division of labor. As a result, the microbial consortium always has a high tolerance to nutritional shocks and shows adaptability to different environments [14]. The microbial consortia screened in our previous research presented high degradation efficiency in the process of degrading alkali lignin (a kind of purified lignin commodity). So, it is of great necessity to explore the application of these lignin degrading microbial consortia in the biomass treatment. Of course, other researchers report some similar studies. For example, Fang et al. screened the microbial consortium from the decaying plant for the treatment of tree trimmings biomass, which can realize the selective degradation of lignin, and this microbial consortium was also composed of fungi and bacteria [15]. Ali et al. investigated the effective bio-pretreatment of sawdust waste utilizing a novel microbial consortium and reported that the capability of a single strain to degrade lignin was much weaker than using a microbial consortium [16].

On the other hand, according to the analysis of the existing research results, compared with other physical and chemical methods, biological pretreatment still has dilemmas in low lignin degradation efficiency and long degradation time, which is not conducive to industrial application. Previous research has shown that it is a promising method to combine biological pretreatment with other pretreatment methods to achieve better treatment effect. Consequently, this research selected microbial consortia with high lignin degradation rate and strong environment adaptability to degrade lignin in three kinds of biomass (eucalyptus root, bagasse and corn straw) and biodegradation process was combined with other physical–chemical method to make up for the shortcomings. The degradation mechanism of lignin can be better explained by analyzing and comparing the degradation of different biomass. The research objects mainly include the degradation efficiency of lignin by the microbial consortium in different biomass under different physical–chemical pretreatment conditions such as steam explosion treatment, the selective degradation ability of the microbial consortium and the cellulase hydrolysis efficiency after the microbial consortium treatment. Meanwhile, the degradation mechanism was discussed and analyzed by examining the changes of morphology and community structure in the process of biomass degradation. The research results aim to provide reference for the efficient utilization of microbial consortium to degrade lignocellulose.

## Materials and methods

### Materials

The original microbial consortia were screened from the wooden relics [13]. Through the preliminary biomass degradation experiment, two microbial consortia J-6 and J-1 with the highest lignin degradation efficiency were selected for this research. According to genomic sequencing analyses, the fungi of J-6 were dominated by Saccharomycetales (98.92%) and the bacteria of J-6 were mainly *Shinella* sp. (47.38%), *Cupriavidus* sp. (29.84%), and *Bosea* sp. (7.96%) and the fungi of J-1 were dominated by Saccharomycetales (98.56%) and the bacteria of J-1 were mainly *Serratia* sp. (95.23%) *and Yersinia* sp. (2.33%) [13]. Species with low relative abundance were not listed. In this experiment, eucalyptus root (Guangxi, China), bagasse (Guangxi, China) and corn straw (Heilongjiang, China), which are common lignocellulosic biomass in China, were selected for the experiments. The content of biomass components is as follows. For eucalyptus root, the lignin content was 26.31 ± 2.94%, cellulose was 40.48 ± 1.95%, and hemicellulose was 18.80 ± 1.54%. For bagasse, the lignin content was 20.76 ± 2.73%, cellulose was 42.95 ± 4.42%, and hemicellulose was 20.82 ± 0.94%. For corn straw, the lignin content was 16.37 ± 3.69%, cellulose was 39.54 ± 5.43%, and hemicellulose was 24.91 ± 4.80%. The biomass materials were screened with a 40–80 mesh sieve after crushing, and dried at 105 °C in an oven for 72 h to prepare for bio-degradation experiments. The biological reagent used in the experiment was purchased from Sigma Company. Other reagents (analytical grade) were purchased from Beijing Chemical Industry Group Co., Ltd.

### Culture conditions

#### Domestication medium

The domestication medium was composed of 2.5 g/L peptone, 1 g/L potassium dihydrogen phosphate, 2.5 g/L sodium chloride, 0.2 g/L MgSO_4_·7H_2_O, 0.1 g/L calcium chloride, 1.6 g/L untreated raw biomass (eucalyptus root, bagasse or corn straw) and 1 mL/L trace element solution. Trace element solution included 0.1 g/L ZnSO_4_ 7H_2_O, 0.1 g/L MnSO_4_·H_2_O, 0.15 g/L CuSO_4_·5H_2_O, 0.16 g/L CoCl_2_·6H_2_O, 0.8 g/L Na_2_MoO_4_ 8H_2_O, 0.02 g/L H_3_BO_3_, and 0.05 g/L NiCl_2_·6H_2_O.

#### Basic medium

The composition of culture medium was as follows: 2.5 g/L (NH_4_)_2_SO_4_, 1 g/L potassium dihydrogen phosphate, 2.5 g/L sodium chloride, 0.2 g/L MgSO_4_·7H_2_O, 0.1 g/L calcium chloride, 0.5 g/L glucose and 1 mL/L trace elements, which were obtained from our previous experiments. Besides, 1 mol/L HCl or NaOH solution was added to the solution to adjust the pH value.

#### Degradation medium

Firstly, 1.6 g/L of biomass (eucalyptus root, bagasse and corn straw) was added to the basic medium to prepare the biomass degradation medium. At the same time, to degrade biomass better, the biomass was pretreated by steam explosion method. Therefore, the biomass under four different treatment conditions (no steam explosion treatment, 1.2 MPa, 1.6 MPa and 2.0 MPa treatment) was added to the basic medium with the same concentration. The concentration of biomass was obtained by optimizing the lignin degradation experiment.

### Biomass pretreatment method

Before steam explosion treatment, the biomass was firstly immersed in water for 10 min, and then the steam explosion equipment (QB-200, Zhengdao Machine Factory, Henan, China) was used for steam pretreatment at the pressure of 1.2 MPa, 1.6 MPa and 2.0 MPa, with the pressure held for 180 s.

### Lignin degradation experiments

The original microbial consortium was transferred to the domestication medium with 10% inoculum volume. Three different domestication media were prepared by three kinds of biomass to acclimate and culture the microbial consortia, respectively. The microbial consortia were cultivated at pH 5, 30 °C and 160 rpm for 14 days for domestication. Then the microbial consortia were cultured in the degradation medium for three cycles, followed by the lignin degradation optimization experiments. The lignin degradation experiment was conducted by changing the culture conditions. By changing the culture conditions, the optimization experiment of lignin degradation in biomass was carried out. In the control group, no microorganisms were added under the same operation mode. The culture conditions changed in the lignin degradation optimization experiment include carbon source (different biomass and different steam explosion pretreatment), pH (4–8) and degradation time (3–28 days). Through the degradation optimization experiment, the best lignin degradation efficiency was obtained and the factors affecting lignin degradation were determined. The degradation medium was placed in a constant-temperature shaker (HS-211B, Shanghai Huanjing Test Equipment Factory), incubated at a temperature of 30 °C and a rotating speed of 180 rpm. After the degradation reaction, the residual biomass was tested and analyzed to judge the degradation of different components (mainly including lignin, cellulose and hemicellulose).

### Analytical methods

#### Determination of three components

The contents of lignin, cellulose and hemicellulose components in biomass were determined according to the NREL method of the U.S. Department of Energy [17]. The biomass dried to constant weight was extracted with the extractor (APLE-3500, Beijing Jitian Equipment Co., Ltd., China). Ultra-pure water and absolute ethanol were selected as the extraction solvents. The extraction temperature was 100 °C, and the rinsing volume was 40% (the volume of the extraction tank was 34 mL). The heat balance and extraction time was 300 s with a final purging process lasting for 60 s. The samples were transferred to glass dishes after extraction, and dried in the oven again. The extracted biomass was then treated by acidolysis, filtration, drying, weighing and other steps according to the standard method [17]. The sugar concentration was determined by high performance liquid chromatograph (HPLC, LC-40D, Shimadzu, Japan) to calculate the content of cellulose and hemicellulose components. The SV was introduced to characterize the selective degradation efficiency of lignin during the biomass degradation process by microbial consortia [18].

#### Calculation of degradation efficiency

The degradation efficiency of the three components can be calculated by: Degradation efficiency (%) = (initial content * initial biomass mass—content after treatment * biomass mass after treatment)/initial content * initial biomass mass.

And according to NERL method [17], the three components content can be calculated by:

$${\text{Cellulose content }}\left( \% \right)\, = \,\left( {{\text{C}}_{{\text{c}} - {6}} *{ 87 }* \, 0.{9}} \right)/\left( {{1}000 \, *{\text{ biomass mass}}} \right) \, *{ 1}00\%$$

$${\text{Hemicellulose content }}\left( \% \right)\, = \,\left( {{\text{C}}_{{\text{c}} - {5}} *{87}*0.{88}} \right)/\left( {{1}000 \, *{\text{ biomass mass}}} \right) \, *{ 1}00\%$$

Lignin content (%) = acid soluble lignin content (%) + acid insoluble lignin content (%).where *C*_c-6_ (g/L) is the hexoses (glucose, galactose, and mannose) concentration by acid hydrolysis and *C*_c-5_ (g/L) is the pentoses (xylose and arabinose) concentration by acid hydrolysis.

SV = lignin degradation efficiency/cellulose degradation efficiency.

#### Determination of saccharification efficiency

According to the literature [19], 20 FPU/g of cellulase (CAS: 9012-54-8) was added to assay the saccharification efficiency of the biomass before and after the lignin degradation treatment. After the saccharification reaction, samples were centrifugated with high-speed centrifuge (Sorvall ST8, ThermoFisher) at 5000 rpm for 3 min, and the reducing sugar content was determined simultaneously by HPLC and DNS method. The saccharification efficiency was calculated as follows: saccharification efficiency (or the yield of enzymatic hydrolysis) % = Mass of reducing sugar produced *0.9/mass of cellulose and hemicellulose in the sample.

#### Morphological detection

The microstructure of the biomass samples was characterized by a scanning electron microscope (SEM, SUPRA55, Carl Zeiss AG, Germany). The biomass samples were treated into fine filaments and then glued to the SEM sample holder. After spraying gold, the morphological changes of biomass were analyzed at an accelerating voltage of 5.0 kV. The fiber crystallinity test was conducted using an X-ray diffractometer (XRD-6000, Shimadzu, Japan) according to the previous research [18].

#### Community structure detection

To ensure the uniformity of results, the kits and primer pairs used in this study were the same as those in our previous study [13]. Microbial community genomic DNA was extracted from the samples using the E.Z.N.A.^®^ soil DNA Kit (Omega Bio-Tek, USA). The primer pair 515F (5ʹ-GTGCCAGCMGCCGCGG-3ʹ) and 806R (5ʹ-GGACTACHVGGGTWTCTAAT-3ʹ) was used to amplify bacterial 16S rRNA genes in the community. The primer pair ITS1F (5ʹ-CTTGGTCATTTAGAGGAAGTAA-3ʹ) and ITS2R (5ʹ- GCTGCGTTCTTCATCGATGC-3ʹ) was used to amplify fungal ITS genes. The purified amplicons were sequenced on MiSeq PE300 platform/NovaSeq PE250 platform (Illumina, USA). The data processing method of gene sequence was based on the literature [20].

### Statistical methods

All the above experiments were repeated 3 times and the average data were reported. One-way ANOVA was used to detect any significant differences in the results (Tukey’s test, *p* < 0.05).

## Results and discussion

### Lignin degradation in biomass

In our previous research, it has been confirmed that the microbial degradation process of lignin was mainly related to carbon source composition, degradation pH, and degradation time. Therefore, this research mainly investigates the effects of these factors on the degradation of lignin in biomass.

At the same time, our previous experiments also showed that the microorganisms of the microbial consortium had a certain demand for oxygen during the growth and the lignin utilization, because shaking incubation can achieve higher degradation efficiency. Accordingly, shaking incubation was performed in this research and the results showed that temperature had a negligible effect on lignin degradation efficiency within the range of 160–200 rpm. In addition, degradation experiments at different temperatures were also carried out. The results demonstrated that temperature also had a negligible effect on lignin degradation within the temperature range of 25–35 °C. Because the rotation speed and temperature both had little effect on the lignin degradation, in the subsequent experiments, the degradation temperature was 30 °C, and the rotation speed was 180 r/min. And lignin was not degraded in the control group without adding microorganisms.

#### Effect of pH on microbial consortium degradation

**Fig. 1:**
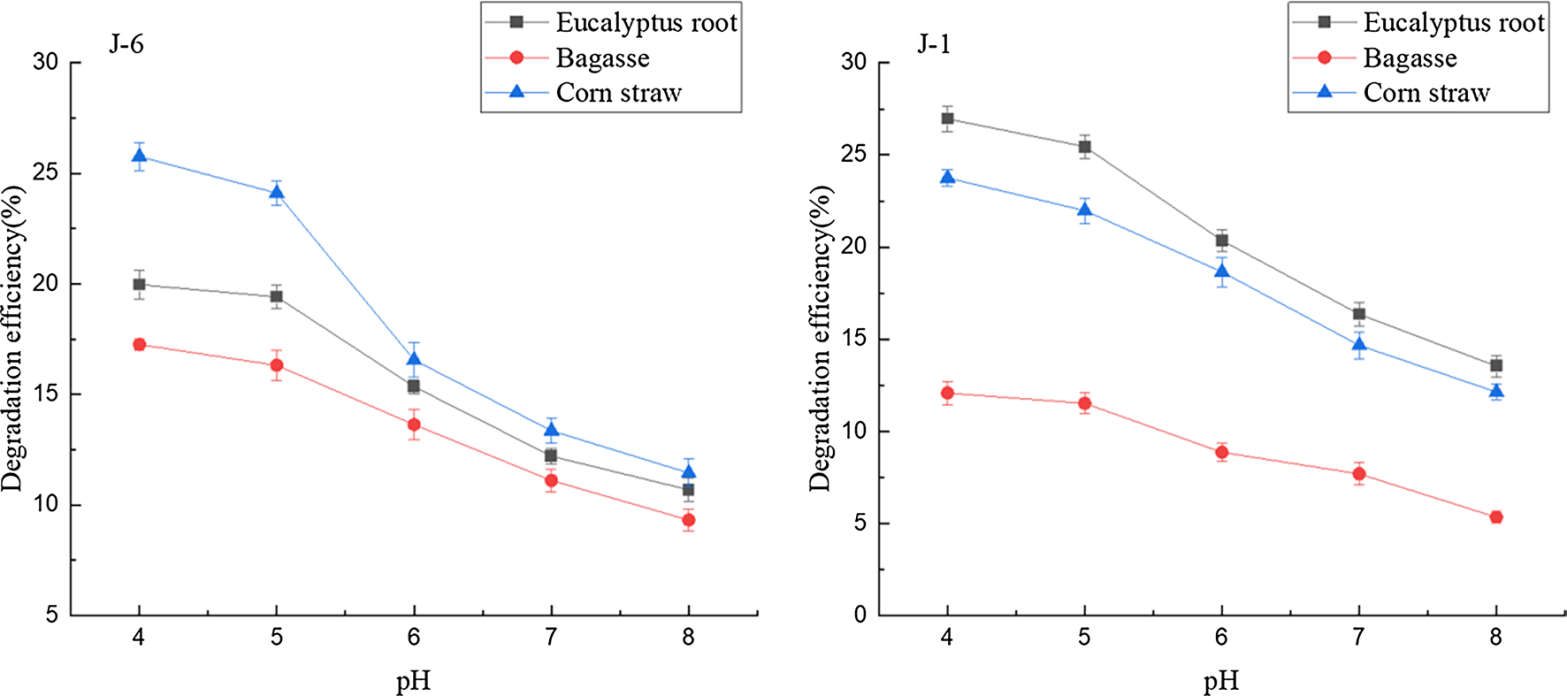
Effect of initial pH on lignin degradation in biomass by microbial consortia

When lignin is degraded by microorganisms, the pH value of the culture medium is an important factor affecting the degradation efficiency. However, the most suitable pH value for lignin degradation by each microorganism is usually very different. For example, it was reported that the optimal pH value for *Aneuribacillus aneurilyticus* to degrade lignin was 7.6 [21]. Our previous research showed that J-6 and J-1 reached the highest efficiency of degrading purified lignin under acidic condition [13]. The efficiency of J-6 and J-1 in degrading lignin in biomass under different pH conditions is pictured in Fig. 1. The raw materials used in the experiment were three kinds of biomass without steam explosion treatment, and the biodegradation time was 14 days. The results showed that when the pH was 4.0–8.0, the microbial consortia could grow and degrade lignin. When the pH was 4.0–5.0, the lignin degradation efficiency was the highest. In the subsequent experiments, pH was 4. Through one-way ANOVA, pH was not a significant factor affecting lignin degradation in biomass, which was consistent with our previous research. Under acidic conditions, the lignin degradation efficiency was higher, which may be related to the microbial community composition and enzyme activity of lignin-degrading microorganisms. According to our previous study [13], under the condition that the fungi were mainly composed of Saccharomycetales, acidic condition was more suitable for the degradation of lignin. The Saccharomycetales adapted to the lignin substrate media and may have promoted lignin degradation. To determine the effect of yeast on the activity of lignin-degrading enzymes, J-6 was treated with nystatin to inhibit yeast activity. The results indicated that yeast in the microbial consortium improved the enzyme activity of lignin-degrading enzymes and thus increased the lignin degradation efficiency. At the same time, the lignin-degrading enzyme activity has been reported to be higher under acidic conditions [22]. In addition, lignin in biomass materials might be demethylated under acidic conditions and the increase of phenolic hydroxyl content might improve the degradation efficiency of lignin [23], which also verified the experimental results of this research.

#### Effect of steam explosion treatment on microbial consortium degradation

**Fig. 2:**
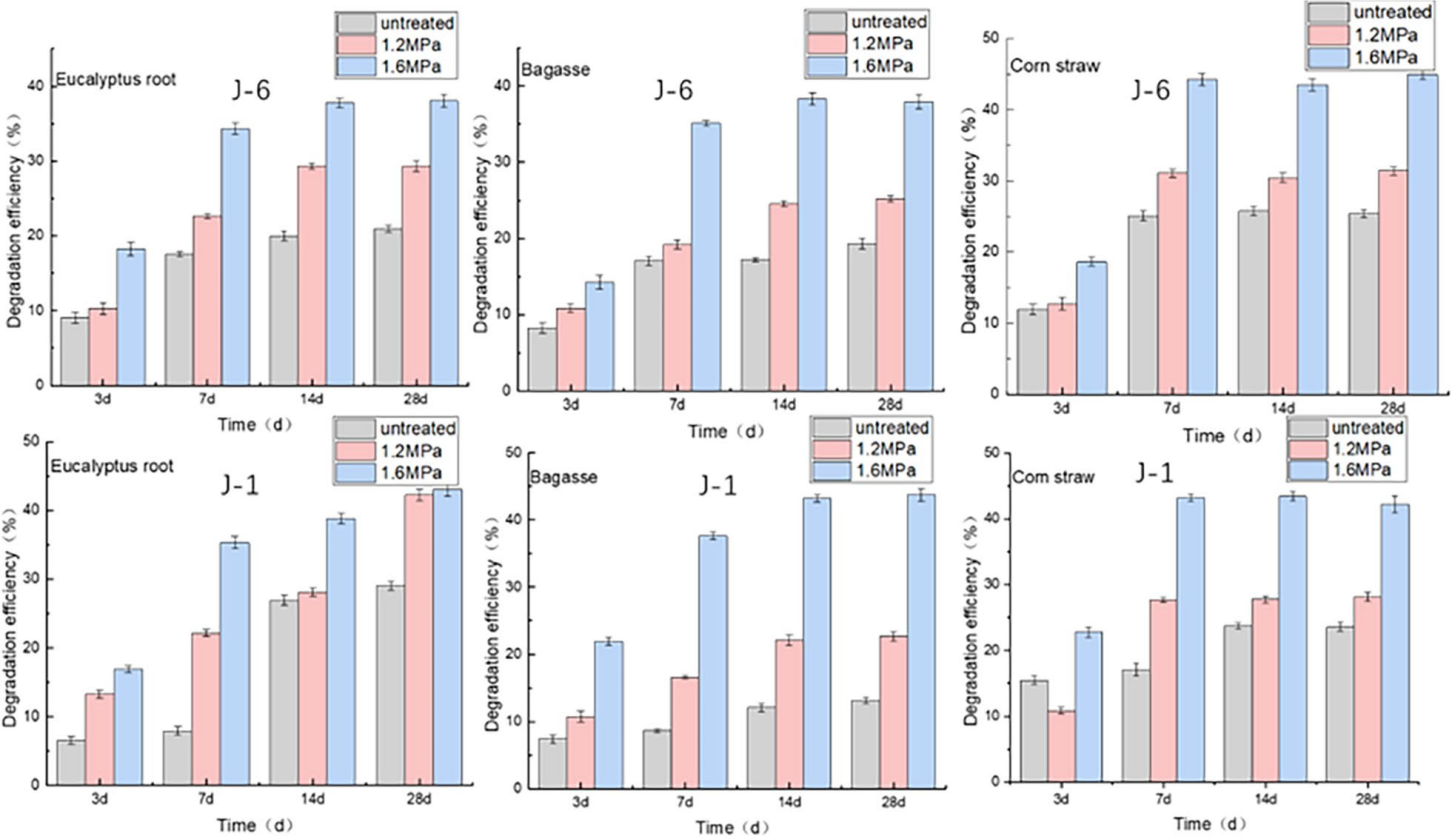
Lignin degradation in biomass under different treatment conditions by microbial consortia (Degradation temperature: 30 °C; Rotation speed: 180 r/min; pH: 4)

Steam explosion is the most commonly used biomass pretreatment technology at present. It utilizes high-temperature and high-pressure steam to handle materials and instantly release pressure, so as to realize component separation and internal structure change of materials [24, 25]. Through researching the relationship between the internal structure of biomass and effective utilization, Asada et al. found that the pore distribution of softwood biomass could be changed by steam explosion pretreatment [26]. Silva et al. used bagasse as raw material and pretreated it with water and the experimental results showed that 12.86% of lignin and 14.89% of hemicellulose were dissolved by steam explosion treatment, while the cellulose retention efficiency was as high as 97.5% [27]. The structural changes caused by steam explosion treatment may have a beneficial effect on the lignin degradation by the microbial consortium. This research proposes a method combining steam explosion pretreatment with lignin bio-degrading treatment. The steam explosion treatment could provide better attachment and living place for microorganisms, and improve the lignin degradation efficiency by microbial consortium. This combined treatment method can also reduce the by-products produced in the pretreatment process, thereby improving the enzymatic hydrolysis efficiency in the subsequent treatment process. In this experiment, three different pressure values (1.2 MPa, 1.6 MPa and 2.0 MPa) were selected for steam explosion pretreatment of biomass. The degradation efficiency of lignin was investigated under the above three pressure conditions and the condition without steam explosion treatment. The experimental results are shown in Fig. 2. It should be noted that 2.0 MPa treatment has high energy consumption and poor pretreatment results, which was not chosen in the subsequent experiments.

The experimental results revealed that the lignin degradation efficiency of the three kinds of biomass treated by steam explosion was improved compared with that of the untreated biomass, especially under the pressure of 1.6 MPa. After 1.6 MPa treatment, the lignin degradation efficiency of eucalyptus root reached 34.36% on the 7th days by J-6 and 35.35% on the 7th days by J-1 microbial consortium treatment, which was significantly higher than that of the biomass without explosion treatment. The lignin degradation efficiency of bagasse was 37.61% after the combined treatment of steam explosion and J-1 microbial biotreatment for 7 days. The lignin degradation efficiency of corn straw processed by steam explosion and then treated by J-6 microbial community for only 7 days reached 44.24%. However, Zhang used mixed strains to degrade corn straw (without steam explosion), with the maximum lignin degradation efficiency reaching 29.1% [28]. Zhen et al. also reported that steam explosion pretreatment can improve the microbial degradation efficiency of lignocellulose biomass [29]. The results of our experiment were also consistent with their conclusion. Therefore, steam explosion can provide better conditions for subsequent microbial degradation by changing the material structure, and its mechanism will be discussed in the follow-up part.

In addition, after the steam explosion treatment in this study, small amount of degradation of the hemicellulose and lignin occurred and cellulose remains essentially the same as before treatment which was consistent with the results of the reference [30]. Taking 1.6 MPa treatment as an example, the degradation efficiency of lignin in biomass after explosion was only 7.72% for eucalyptus roots, 5.31% for bagasse, and 8.83% for corn straw. However, after the combined treatment, the lignin degradation efficiency of the corn straw could reach more than 40%. According to other literature, the lignin degradation efficiency of corn straw optimized by steam explosion treatment can reach about 24% [31], which was much lower than that of our biological method. It was further confirmed that the steam explosion combined with microbial consortium degradation could greatly improve the lignin degradation efficiency.

#### Effect of degradation time on microbial consortium degradation

The effect of degradation time on microbial consortium degradation is also illustrated in Fig. 2. The biomass after steam explosion reached higher degradation efficiency within 7 days after adding two microbial consortia. The degradation efficiency of bagasse and eucalyptus root reached more than 30% in 7 days, and that of corn straw reached more than 40%. This demonstrated the advantages of biological method in the treatment of straw biomass. After 14 days, the degradation efficiency increased slightly with the extension of degradation time. Among them, the highest lignin degradation efficiency of corn straw biomass reached 44.24%. The optimal degradation efficiency was reached in the biomass under 1.6 MPa treatment. According to other literatures, Zhang et al. used the mixture of *Trichoderma koningiopsis* and *Phanerochaete chrysosporium* to degrade corn straw and after 11 days of treatment, the lignin degradation efficiency was 28.87% [32]. Zhu et al. chose *Trametes versicolor* as the experimental strain for lignin degradation. After 21 days of culture, the lignin degradation efficiency of straw reached 34.8% [33]. In contrast, the microbial consortium used in this experiment showed strong degradation ability to lignin, and the degradation time was greatly shortened, providing a novel microbial resource and research direction for the degradation of lignin. The lignin-degrading ability of the microbial consortium was related to the activity of lignin-degrading enzymes. Our previous studies showed that both J-1 and J-6 can produce three different lignin-degrading enzymes: laccase, LiP (Linin Peroxidase) and MnP (Manganese Peroxidase) [13].

In summary, the main defects of the current microbial pretreatment methods for lignocellulose materials are slow reaction speed and difficulty in industrial application. The combination of biological method and physical–chemical method in this research may provide a better idea. Steam explosion and microbial consortium degradation can produce a coupling effect. The combined treatment method achieved high lignin degradation efficiency for three different types of biomass raw materials of eucalyptus root, bagasse and corn straw. In the following content, the results of morphological analysis, enzymatic hydrolysis performance and microbial consortium composition and structure will be discussed in detail to explain the corresponding conclusions.

### Selective degradation of lignin by microbial consortia

The microbial consortia used in this research was obtained from decayed wooden antiques. Cellulose, hemicellulose and other compounds in wooden relics that are easy to be used by microorganisms are largely decomposed during long-term burial, thus forming a lignin rich environment (lignin content > 60%), which can create good living conditions for lignin degrading microorganisms [34]. The microbial consortia were mainly screened according to the ability to produce lignin-degrading enzymes. Therefore, they have high lignin degradation capacity. For better application in biomass degradation, the ability to selectively degrade lignin is required to avoid the degradation of other effective components in lignocellulose. The selective degradation ability of the microbial consortia was investigated, and the SVs were calculated. The results are presented in Table 1.

**Table 1:** Lignin degradation SV values of different biomass by microbial consortia

	SV		
	Bagasse	Corn straw	Eucalyptus root
Without steam explosion treatment (J-6)	2.12	3.02	2.07
1.2 MPa steam explosion treatment(J-6)	2.75	3.23	2.39
1.6 MPa steam explosion treatment(J-6)	2.68	3.59	2.35
Without steam explosion treatment ((J-1)	2.33	1.89	2.46
1.2 MPa steam explosion treatment(J-1)	2.56	2.11	2.77
1.6 MPa steam explosion treatment(J-1)	2.85	2.33	2.84

The SV can be calculated by the ratio of lignin degradation efficiency to degradation efficiency of cellulose. The higher the value, the better the selective degradation ability of microorganisms to lignin is. The SV of screened DM-1 microbial consortium was 2.78 [15]. In this research, the biomass materials with high lignin degradation efficiency were tested. It was found that the SV of various biomass after 1.6 MPa steam explosion treatment was significantly higher than this value. The SV of J-6 was up to 3.59. And the SV of J-1 was up to 2.85. The selective degradation ability of J-6 was relatively strong. Meanwhile, the composition and structure of the biomass materials were also different, which also might lead to differences in the selective degradation ability of the microorganisms. The stable structure of eucalyptus root may be the reason for its weaker selective degradation ability compared with other kinds of biomass materials. The experimental results showed that the microbial consortia could selectively degrade lignin in the presence of cellulose. Simultaneously, the composition of microbial consortium might be closely related to the selective degradation ability, which needs to be further explored.

### Effect of microbial consortium treatment on enzymatic hydrolysis

**Fig. 3:**
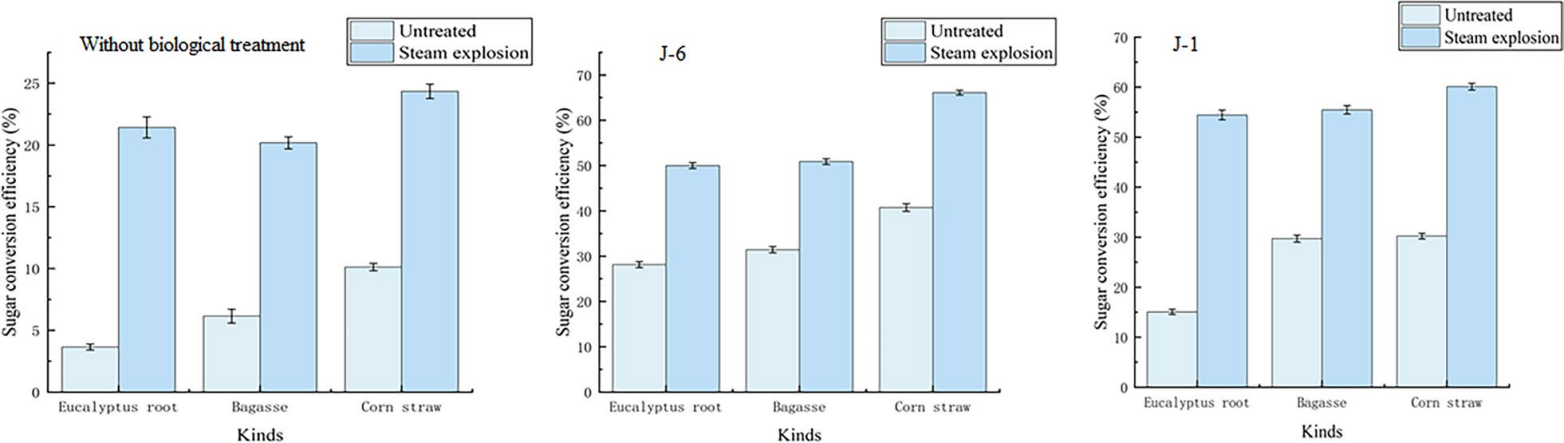
Saccharification efficiency of biomass under different treatment conditions

The literature reported that the pretreatment of lignocellulose materials by lignin-degrading microorganisms can significantly improve the saccharification efficiency of cellulase hydrolysis [35]. By the treatment of lignin-degrading microorganisms, the original structure of lignocellulose can be broken and the content of lignin can be reduced, thereby increasing the cellulase action area [36]. The saccharification process can be promoted due to the weakened inhibition of cellulose activity. In this research, the biomass treated by microbial consortium was enzymatically hydrolyzed, and the result was compared with the untreated biomass. The change of saccharification efficiency is shown in Fig. 3. The results illustrated that the saccharification efficiency after biological treatment was significantly improved and positively correlated with the degradation efficiency of lignin. Lignin is considered a major obstacle to the enzymatic hydrolysis of cellulose, as it is closely associated with cellulose microfibrils [37] and microbial consortium can enhance the enzymatic digestion of lignocellulosic biomass by changing the chemical composition through degradation of lignin. Although some previous studies have shown that the phenols produced by pretreatment before lignin degradation usually inhibit enzymatic hydrolysis [24, 36], no significant inhibition was found in the treatment by microbial consortium. Compared with wood, corn straw has lower lignin content and looser structure, which is more suitable for biological pretreatment. Without steam explosion treatment, only biological treatment can achieve better results, and the total saccharification efficiency of direct biological pretreatment can also reach 40.76%.

Furthermore, the results showed that the cellulase hydrolysis efficiency after steam explosion treatment was also significantly improved. The structure of lignocellulose was destroyed due to its own expansion during the steam explosion process [38]. Thus, the pretreatment of eucalyptus biomass by the combined treatment can gain better pretreatment effect. Moreover, the treatment time of the microbial consortium in this research was only 7 days, which was less than the biological pretreatment time reported in many literatures. For example, Keller et al. reported the treatment time of the corn straw pretreated by *C. stercoreus* was 29 days and the saccharification efficiency reached 36% when corn straw was hydrolyzed under the condition of enzyme dosage of 60 FPU/g [39]. At the same time, the treatment time of the wood biomass with biological pretreatment reported in the literature was always very long, even up to 120 days [40]. In this research, the enzymatic hydrolysis efficiency of eucalyptus root after 7 days of microbial consortium pretreatment was significantly improved, which can reflect the advantages of microbial consortium treatment in this research.

### Morphological analysis

The experimental results of lignin degradation efficiency and the effect of microbial degradation pretreatment on biomass saccharification efficiency can confirm that both steam explosion treatment and microbial treatment may affect the structure of biomass, and the structures and morphologies of different biomass are different.

**Fig. 4:**
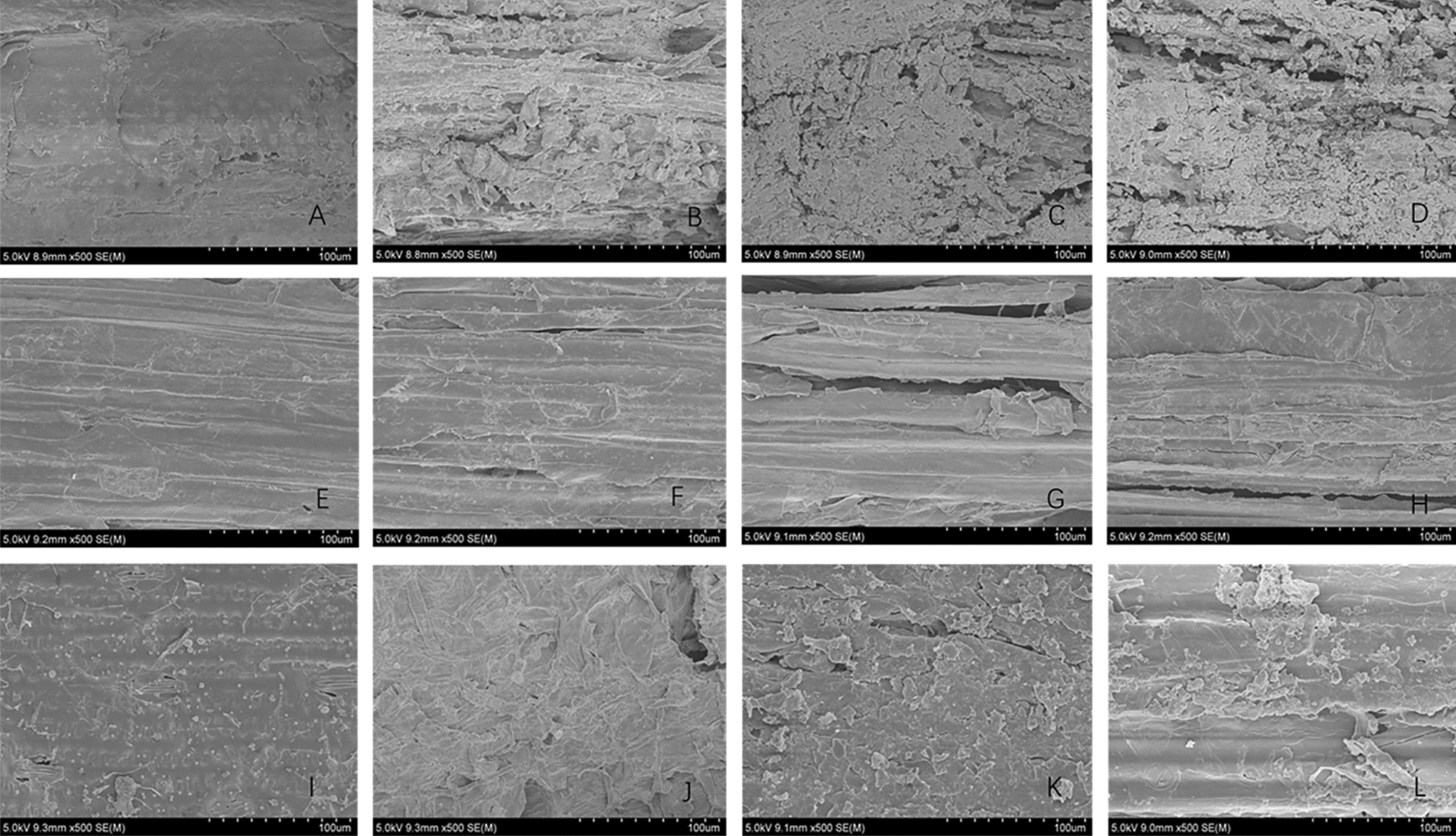
SEM morphology of biomass under different treatment conditions. (**A**: Eucalyptus root; **B**: Eucalyptus root after steam explosion treatment; **C**: Eucalyptus root after steam explosion- J-6 combined treatment; **D**: Eucalyptus root after steam explosion- J-1 combined treatment; **E**: bagasse; **F**: bagasse after steam explosion treatment; **G**: bagasse after steam explosion-J-6 combined treatment; **H**: bagasse after steam explosion-J-1 combined treatment; **I**: corn straw; **J**: corn straw after steam explosion treatment; **K**: corn straw after steam explosion- J-6 combined treatment; **L**: corn straw after steam explosion- J-1 combined treatment)

In this experiment, SEM was used to obtain the morphology of three kinds of biomass (Fig. 4) and to observe the surface structure of different materials. Then the effects of various pretreatment methods on the morphology were evaluated. Further, we analyzed the mechanism of steam explosion combined with microbial treatment to achieve efficient lignin degradation and to improve the enzymatic hydrolysis efficiency. The three kinds of original biomass all presented a relatively smooth surface structure, but the density of the material surface was different. The density of eucalyptus root was higher, and the structure was more stable. The density of corn straw material was relatively lower. The surface of the biomass after steam explosion treatment was loose, and the fiber structure was destroyed, resulting in some pores especially that the eucalyptus root displayed significant structural damage. According to literature [41], lignin-degrading microorganisms easily adhere and grow in the cracks and pores of biomass materials, and further secrete enzymes to destroy biomass structure and produce macrospores. Therefore, steam explosion pretreatment was conducive to the microbial consortium growth in the biomass materials and contributed to the degradation of lignin in biomass. SEM results also demonstrated that the structure of eucalyptus root was partially damaged and perforated after microbial consortium treatment. The bagasse had large cracks after microbial consortium treatment. Moreover, fragmentation appeared on the surface of corn straw after microbial consortium treatment. From the morphological analysis, it could be inferred that the destruction degree of the biomass materials may show a certain correlation with the lignin degradation efficiency. Morphological changes are also related to subsequent enzymatic hydrolysis efficiency and the increase of sample porosity and the destruction of structure could increase the accessibility of enzymatic hydrolysis [26, 37], which verified our previous research results.

In addition, researchers have found that the crystallinity of cellulose has a certain impact on the efficiency of enzymatic hydrolysis [42, 43]. In this research, the crystallinity of cellulose decreased after steam explosion, and the average reduction of the three kinds of biomass was 10.3%. However, the crystallinity of cellulose was not changed significantly after the pretreatment by the lignin degradation microorganisms. Therefore, the microbial consortium showed strong selective degradation ability. The biodegradation process by the microbial consortium was mainly aimed at the degradation of lignin in biomass materials, and had no obvious effect on the cellulose crystallization zone. Therefore, if the pretreatment method of steam explosion combined with microbial consortium biodegradation was adopted, the crystallinity of cellulose can be reduced and the lignin can be removed. Meanwhile, the porosity of raw biomass materials can be improved to increase the effective contact area between cellulase and cellulose, and the amount of inhibitor in the enzymatic hydrolysis system can be reduced.

### Microbial consortium structure analysis

The above experiments confirmed that the J-6 microbial consortium had strong adaptability to different environments and high degradation efficiency of lignin in biomass. Other research showed that the main performance of the strong adaptability would be that the community structure of the microbial consortium can change with environmental conditions [44]. Through the analysis of microbial genomic sequencing, we studied the variation of community structure and dominant bacteria in the process of biomass degradation and discussed the mechanism of lignin degradation by microbial consortium to provide data reference for the future efficient utilization of microbial synergistic degradation of lignin.

#### Microbial community composition

Genomic sequencing analyses indicated that there was no significant difference in the fungal community composition of the microbial consortia of different systems, which was nearly the same as that of the original microbial consortia. Fungi of the microbial consortium were mainly composed of Saccharomycetales. This also confirmed the conclusion of 3.1.2. In addition, according to our previous research [13], the lignin degradation enzyme activities of J-1 and J-6 were closer, which may be related to the similar fungal composition of J-1 and J-6. Fungi play an important role in lignin degradation system. For example, Saccharomycetales in the microbial consortium improved the enzyme activity of lignin-degrading enzymes and thus increased the lignin degradation efficiency [13, 45]. The stability of fungal community may have an important relationship with the maintenance of the lignin degradation ability of different systems.

**Fig. 5:**
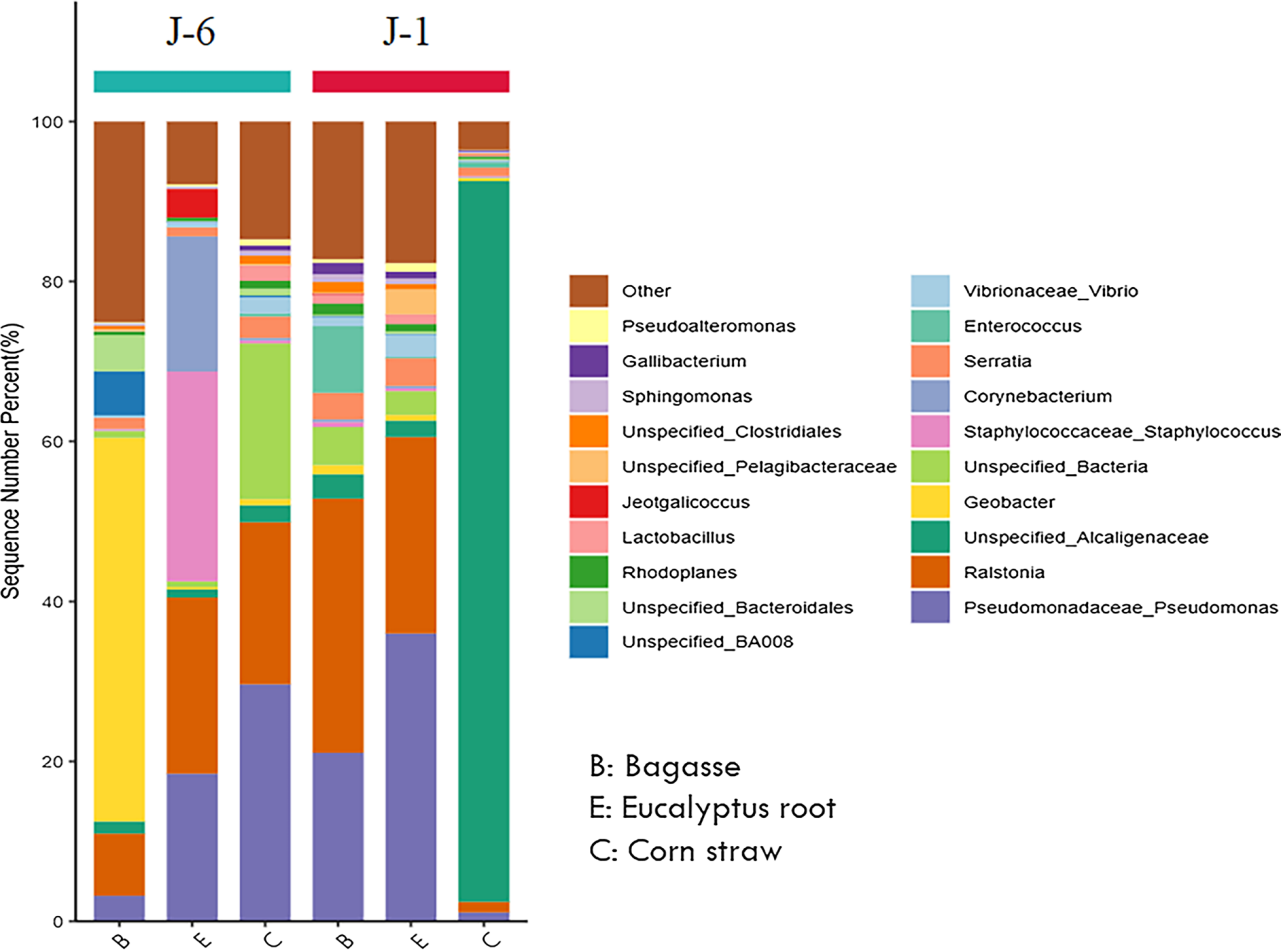
Bacterial community composition of different biomass degradation systems

However, the bacterial composition of J-6 and J-1 microbial consortium was changed significantly in different systems (Fig. 5), and was different from the original J-6 microbial consortium. The proportion of dominant bacteria *shinella* sp. in original J-6 and *Serratia* sp. in original J-1 decreased significantly. In addition, in different biomass systems, the bacterial composition was also obviously different. J-6 in eucalyptus root system was mainly composed of Staphylococcaceae (26.32%), *Ralstonia* (22.12%) and Pseudomonadaceae (18.55%). In the bagasse system, J-6 was mainly composed of *Geobacter* (48.25%) and *Ralstonia* (7.81%). In the corn straw system, J-6 was mainly composed of Pseudomonadaceae (30.04%) and *Ralstonia* (20.53%). J-1 in Eucalyptus root system was mainly composed of 24.65% *Ralstonia* and 36.33% Pseudomonadaceae. In bagasse system, J-1 was mainly composed of 32.05% *Ralstonia* and 21.24% Pseudomonadaceae. In the corn straw system, J-1 was mainly composed of 90.15% Alcaligenaceae.

Additionally, there are certain similarities in bacterial composition among different biomass degradation systems. For example, *Ralstonia* and Pseudomonadaceae are abundant in eucalyptus root system. The bagasse biomass also contains a large number of these two kinds of bacteria, indicating that *Ralstonia* and Pseudomonadaceae can play an important role in the biomass system for lignin degradation. These two kinds of bacteria are also present in the biomass degradation microbial consortia in other literatures, such as the corn straw degradation microbial consortium GF-20 [46] and the wheat straw degradation microbial consortium [47]. According to other literature [48], Pseudomonadaceae is very common in the degradation process of lignocellulose in nature, and has strong adaptability to biomass degradation systems. The composition of community structure should be closely related to degradation behavior of microbial consortium.

There were great differences in bacterial composition between the original J-6 and J-1 microbial consortium, and there were also obvious differences in community structure between J-6 and J-1 in biomass degradation system. Although both of them showed high lignin degradation effect in the biomass degradation system, there were some differences in the efficiency of selective degradation of lignin. The selective degradation ability of J-6 was stronger than that of J-1, and the main difference in the composition of the community structure between J-6 and J-1 was that the proportion of Alcaligenaceae in J-1 was much higher. According to other literature [5, 45], Alcaligenaceae is very common in the degradation process of lignocellulose in nature, and has strong adaptability to biomass degradation systems. At the same time, it could cooperate with other microorganisms such as cellulose degrading bacterium *Clostridium* sp. to promote the decomposition of biomass [5]. This again showed that the composition of community structure should be closely related to degradation behavior of microbial consortium. In addition, the composition of bacteria in the microbial consortium was also related to the types of the lignin degradation products. Our previous study showed that the lignin degradation process of J-6 was more complete and the product structure was simpler than that of J-1 [13].

#### Diversity analysis of community structure

**Fig. 6:**
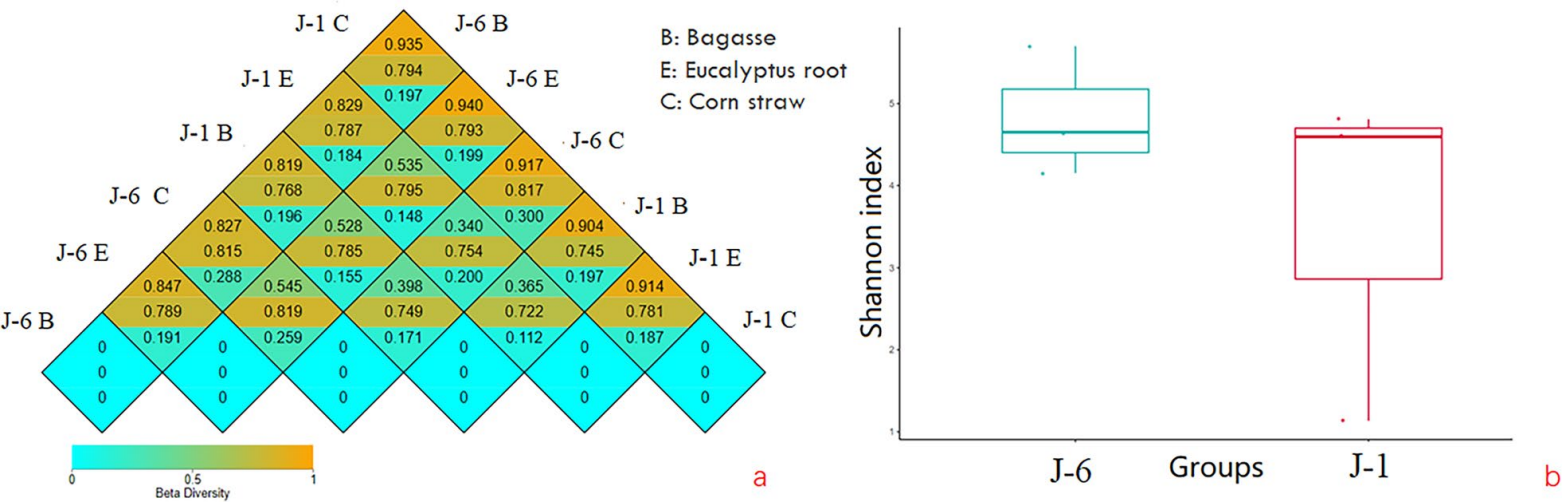
Bacterial community diversity of different biomass degradation systems

Bray Curtis, weighted UniFrac and unweighted UniFrac distances was calculated by the OTU abundance information of samples to evaluate the differences in microbial community structure. Bray Curtis distance is the most commonly used indicator to reflect the differences between communities, and only the abundance information of species is considered. The unweighted UniFrac distance is the distance between samples calculated based on the phylogenetic relationship of species. The weighted UniFrac distance is the distance between samples obtained by combining the abundance information of OTU and the phylogenetic relationship. Unweighted UniFrac distance is more sensitive to rare species, while Bray Curtis and weighted UniFrac distance are more sensitive to species with higher abundance. As beta diversity distance, weighted UniFrac distance, unweighted UniFrac distance and Bray Curtis distance are indicators to measure the dissimilarity coefficient between the two samples. The smaller the value, the smaller the difference between the two samples in species diversity [49, 50]. Figure 6a shows the heat maps of the above three distances between different samples. The results showed that in the J-6 degradation system, there were great differences between different biomass, especially the Bray Curtis distance and the weighted UniFrac distance, which considered the species abundance information were far away, indicating that there were great differences in species abundance in different biomass. But the unweighted UniFrac distance of bagasse and Eucalyptus root was closer than that of bagasse and corn straw indicating that in the phylogenetic relationship of species, the genetic distance between the microbial consortia of sugarcane system and eucalyptus root system was closer. The lignin degradation of eucalyptus root degradation system and bagasse degradation system were also relatively similar according to previous studies. Also, the J-1 degradation system was similar with the J-6 degradation system, which verified that the bagasse degradation system was close to the eucalyptus root degradation system.

Alpha diversity index is an analysis of species diversity in a sample, including two factors: richness and evenness of species composition in the sample. It usually uses ObservedOTU, Shannon and Faith’s Phylogenetic Diversity to evaluate the species diversity of a sample. The higher the index, the more complex the diversity of the sample is [51]. In this study, Shannon index was used to evaluate species diversity. The result is shown in Fig. 6b. The bacterial community diversity of J-6 was still higher than J-1, showing no difference with the original microbial consortium [13].

## Conclusion

Converting lignocellulose waste biomass into high-value products such as biofuel ethanol can not only effectively alleviate the energy shortage, but also be of great significance to achieve the goal of “double carbon”. Lignin structure is stable and difficult to be degraded, which is the main barrier for lignocellulose to achieve high-value transformation. Biological degradation of lignin has low energy consumption, strong safety and good prospects, but the degradation time is long, which affects the industrial application. In this study, the treatment method of biomass steam explosion combined with microbial biodegradation was proposed, which can not only effectively improves the degradation efficiency of lignin, shorten the microbial treatment time, but also greatly improves the enzymatic hydrolysis efficiency of subsequent treatment.

The obtained microbial consortia J-6 and J-1 with high lignin degradation rate and strong environment adaptability were selected to degrade lignin in three kinds of biomass (Eucalyptus root, bagasse and corn straw). The results showed that the lignin degradation efficiency of bagasse and eucalyptus root can reach more than 30% in 7 days, and that of corn straw can reach more than 40% with the steam explosion combined with microbial consortium biodegradation. The selective degradation ability of J-6 and J-1 was also studied. The experimental results showed that in the presence of cellulose and hemicellulose, J-6 and J-1 could selectively degrade lignin. After steam explosion combined with microbial treatment, the enzymatic sugar conversion efficiency of biomass materials also increased significantly.

The above research results were also verified by SEM, fiber crystallinity of biomass materials and microbial consortium structure analysis. It could be inferred that the destruction degree of the biomass materials may show a certain correlation with the lignin degradation efficiency. The increase of sample porosity and the destruction of structure could increase the accessibility of enzymatic hydrolysis. And the crystallinity of cellulose decreased after steam explosion, however, the crystallinity of cellulose was not changed significantly after the pretreatment by the lignin degradation microorganisms. In addition, according to genomic sequencing analyses, the fungal community composition of the microbial consortia in different systems was not significantly different, and was nearly the same as that of the original microbial consortia. But the bacterial composition of J-6/J-1 microbial consortium was changed in different systems. *Ralstonia* and Pseudomonadaceae can play an important role in the biomass system for lignin degradation. The microbial consortium had strong adaptability in different degradation systems.

The steam explosion combined with microbial consortium degradation of lignin pretreatment technology in this study can help waste biomass create higher value-added products. It was also proved that the collaborative degradation of lignin by microbial consortium could overcame the problem of degradation by the single strain. This research also provided a reference for exploring the mechanism of lignin degradation and microorganisms. In the future, the change of lignin degradation products and the mechanism of microbial interaction during degradation will also be studied.

## Data Availability

Most of the recorded data are available in the manuscript. On reasonable request, the corresponding author can provide required data and material.
